# Pharmacokinetics of Oral and Inhaled Terbutaline after Exercise in Trained Men

**DOI:** 10.3389/fphar.2016.00150

**Published:** 2016-06-10

**Authors:** Anders Dyreborg, Nanna Krogh, Vibeke Backer, Sebastian Rzeppa, Peter Hemmersbach, Morten Hostrup

**Affiliations:** ^1^Respiratory Research Unit, Bispebjerg HospitalCopenhagen, Denmark; ^2^IOC Sports MedicineCopenhagen, Denmark; ^3^Norwegian Doping Control Laboratory, Oslo University HospitalOslo, Norway; ^4^Department of Nutrition, Exercise, and Sports, University of CopenhagenCopenhagen, Denmark

**Keywords:** β2-agonist, pharmacology, doping analysis, asthma, WADA

## Abstract

**Aim:** The aim of the study was to investigate pharmacokinetics of terbutaline after oral and inhaled administration in healthy trained male subjects in relation to doping control.

**Methods:** Twelve healthy well-trained young men (27 ±2 years; mean ± SE) underwent two pharmacokinetic trials that compared 10 mg oral terbutaline with 4 mg inhaled dry powder terbutaline. During each trial, subjects performed 90 min of bike ergometer exercise at 65% of maximal oxygen consumption. Blood (0–4 h) and urine (0–24 h) samples were collected before and after administration of terbutaline. Samples were analyzed for concentrations of terbutaline by high performance liquid chromatography coupled to tandem mass spectrometry (HPLC-MS/MS).

**Results:** Pharmacokinetics differed between the two routes of administration. Serum C_max_ and area under the serum concentration-time curve (AUC) were lower after oral administration compared to inhalation (C_max_: 4.2 ± 0.3 vs. 8.5 ± 0.7 ng/ml, *P* ≤ 0.001; AUC: 422 ± 22 vs. 1308 ± 119 ng/ml × min). Urine concentrations (sum of the free drug and the glucuronide) were lower after oral administration compared to inhalation 2 h (1100 ± 204 vs. 61 ± 10 ng/ml, *P* ≤ 0.05) and 4 h (734 ± 110 vs. 340 ± 48 ng/ml, *P* ≤ 0.001) following administration, whereas concentrations were higher for oral administration than inhalation 12 h following administration (190 ± 41 vs. 399 ± 108 ng/ml, *P* ≤ 0.05). Urine excretion rate was lower after oral administration than inhalation the first 2 h following administration (*P* ≤ 0.001). Systemic bioavailability ratio between the two routes of administration was 3.8:1 (inhaled: oral; *P* ≤ 0.001).

**Conclusion:** Given the higher systemic bioavailability of inhaled terbutaline compared to oral, our results indicate that it is difficult to differentiate allowed inhaled use of terbutaline from prohibited oral ingestion based on urine concentrations in doping control analysis. However given the potential performance enhancing effect of high dose terbutaline, it is essential to establish a limit on the WADA doping list.

## Introduction

The prevalence of asthma and exercise-induced bronchoconstriction is high in the athletic population (Carlsen et al., 2008; Fitch et al., 2008; Price et al., 2014; Couto et al., 2015). In endurance sport, the prevalence has been reported to be as high as 30–50% (Parsons and Mastronarde, 2005; Aavikko and Helenius, 2012) compared to the general population prevalence of ~5% in Western countries (Elers et al., 2012a). Therefore, β2-adrenoceptor agonists (β2-agonists) are commonly prescribed bronchodilators to athletes. Use of β2-agonists in competitive sport is restricted by anti-doping regulations in accordance with the World Anti-Doping Agency's (WADA) list of prohibited substances (The World Anti-Doping Agency, 2015b). The 2016-list of prohibited substances restricts use of all β2-agonists except for therapeutic inhalation of salbutamol, formoterol, and salmeterol (The World Anti-Doping Agency, 2016). WADA has introduced urine thresholds and decision limits for salbutamol and formoterol to discriminate therapeutic inhaled use from prohibited supratherapeutic inhaled and systemic use (The World Anti-Doping Agency Laboratory Comittee, 2014; The World Anti-Doping Agency, 2016). Urine concentrations that exceed 1200 ng/ml for salbutamol and 50 ng/ml for formoterol are presumed not to be intended therapeutic use and is considered an adverse analytical finding (AAF). In 2014, β2-agonists accounted for 4% (122/3079) of AAF reported by WADA accredited doping control laboratories. While several pharmacokinetic studies have made it possible to establish urinary thresholds and decision limits for salbutamol and formoterol (Sporer et al., 2008; Elers et al., 2012a,b; Hostrup et al., 2014a; Haase et al., 2015), no urine threshold or decision limit exists for terbutaline. Of the 122 AAFs involving β2-agonists in 2014, terbutaline accounted for 76%, whereas salbutamol and formoterol only accounted for 12% together (The World Anti-Doping Agency, 2014a).

Terbutaline is commonly prescribed to athletes with asthma and exercise-induced bronchoconstriction in Northern Europe. As of 2016, athletes must obtain a therapeutic use exemption (TUE) to use inhaled terbutaline (The World Anti-Doping Agency, 2015a). Because no urinary threshold and decision limit exist for terbutaline, athletes who have acquired a TUE for terbutaline have an open window for supratherapeutic misuse. This is problematic since high-dose inhalation and oral administration of terbutaline have been shown to increase maximal sprint ability and muscle force (Hostrup et al., 2014a,b; Kalsen et al., 2016). While the pharmacokinetics of terbutaline is well-described (Nyberg, 1984; Nyberg and Kennedy, 1984; Borgström et al., 1989; Schmekel et al., 1992; Elers et al., 2012a), data on its pharmacokinetics in relation to doping control are insufficient. Elers et al. (2012a) observed that urine and serum concentrations were similar after therapeutic inhalation of 2 mg and prohibited oral administration of 10 mg at rest in asthmatic and non-asthmatic men, making it difficult to discriminate therapeutic inhaled use from oral misuse in doping control analysis (Elers et al., 2012a). A limitation of that study in relation to doping control analysis, however, was that only serum and urine concentrations were measured, whereas absorption and excretion of terbutaline were not. Likewise, the administered dose was not the daily maximal recommended dose as stated in the manufacturer's summary of product characteristics nor was it equipotent to the maximal allowed daily dose of salbutamol as stated by The World Anti-Doping Agency (2016). Furthermore, concentrations of terbutaline were measured at rest and not in conjunction with exercise, which is of relevance since asthmatic athletes often inhale their medication prior to training or competition as prophylaxes against bronchoconstriction (McKenzie and Fitch, 2011). Exercise increases metabolism and heat production, both of which may affect uptake, concentration and excretion of β2-agonists. Schmekel et al. (1992) observed that the rate of pulmonary absorption was faster during exercise than at rest. In addition, exercise-induced sweating may affect hydration status and urine specific gravity (USG; Kurdak et al., 2010; Fortney et al., 1988). Currently, threshold values for prohibited substances are adjusted for the specific gravity of urine samples only in terms of endogenous threshold substances (19-norandrosterone, glycerol) when dealing with doping analysis (McKenzie and Fitch, 2011; The World Anti-Doping Agency Laboratory Comittee, 2014; The World Anti-Doping Agency, 2014b). Studies have shown that urine concentrations of β2-agonists are partially related to the specific gravity (Sporer et al., 2008; Hostrup et al., 2012), because concentrated urine samples can lead to a higher concentration of the drug in urine. This may pose a problem as it can lead to a false positive sample. The relationship between the specific gravity of the urine sample and the concentration of β2-agonists, especially after exercise, has therefore to be investigated more comprehensively. Taken together, there is still a need to investigate the pharmacokinetics of terbutaline with a setup applicable for training and exercise with respect to doping control analysis.

The purpose of the present study was to investigate the pharmacokinetics of terbutaline after therapeutic inhalation and prohibited oral ingestion in healthy trained subjects in samples collected during and after 90-min of strenuous cycling. In addition, we investigated the relation between USG of urine samples and concentrations of terbutaline.

## Materials and methods

### Subjects and ethical approval

Fourteen healthy well-trained men were included in the study. Subject characteristics are presented in Table 1. Subjects were non-smokers without chronic diseases. All subjects received detailed written and oral information on the study and gave oral and written consent. The study was performed in accordance with the Helsinki-II-Declaration and was approved by the Regional Ethics Committee of The Capital Region of Denmark (H-2-2014-069) and the Danish Health and Medicines Authority (EudraCT number: 2014-002140-40). The study was monitored in accordance with the GCP-ICH guidelines (Good Clinical Practice; ICH Harmonised Tripartitte Guideline, 1994) in collaboration with the GCP-unit of Copenhagen University Hospital.

**Table 1:** **Subject characteristics (*n* = 12)**.

Age (yrs)	27 ± 2
Height (cm)	183 ± 2
Weight (kg)	79 ± 2
FVC (l)	5.83 ± 0.25
FEV1 (l)	4.83 ± 0.22
FEV1/FVC-ratio	0.82 ± 0.02
VO_2_–max (ml/min/kg)	54 ± 2
Body fat (%)	17 ± 2
Lean Body Mass (kg)	66 ± 2

FVC, Forced Vital Capacity; FEV1, Forced Expiratory Volume in 1 second; VO_2max_, Maximal oxygen consumption. Values are means ± SEM.

### Experimental design

#### Screening procedures

Before the pharmacokinetic trials, subjects underwent a screening at the clinic. At the screening, subjects were examined by a medical doctor and electrocardiography (ECG) was performed (Schiller AT-10 plus, Schiller, Switzerland). Furthermore, subjects' body composition was measured by dual X-ray absorbance (Lunar DEXA-scan, GE Healthcare, United Kingdom) and lung function with a spirometer (EasyOne Spirometer, NDD, Switzerland). Lastly, subjects' maximal oxygen consumption ($\dot{\text{V}}{\text{O}}_{2}\text{max}$) and performance were determined during an incremental bike ergometer (Lode Ergometer, Netherlands) test to exhaustion. Prior to the test, subjects warmed up at 100, 150, and 200 W for 4 min at each workload. The incremental test started at 150 W and increased 30 W every min until exhaustion. Subjects were told to keep a cadence of 80–100 rpm during the test. During the warm-up and incremental test, gas exchange was measured breath-by-breath (Oxycon Pro, CareFusion, CA, United States).

#### Pharmacokinetic intervention

**Figure 1:**
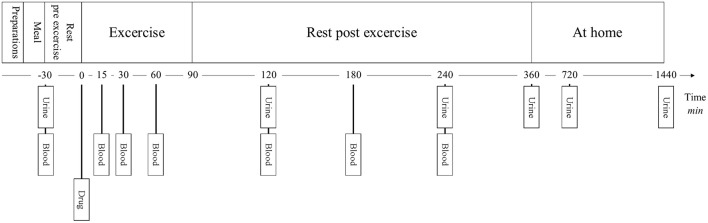
**Experimental overview**.

The pharmacokinetic intervention was an open-labeled crossover study that investigated the pharmacokinetics of terbutaline after dry powder inhalation of 4 mg (Bricanyl Turbohaler, AstraZeneca, Denmark) and oral ingestion of 10 mg (Bricanyl Retard, AstraZeneca, Denmark) in healthy trained men that performed exercise (Figure 1). The administered dosage of inhaled terbutaline did not exceed the maximal daily-recommended dose of 6 mg as stated by the manufacturer. Furthermore, the used dose of 4 mg terbutaline is equipotent to the maximal daily-allowed dose of salbutamol as stated by The World Anti-Doping Agency (2016). The administered dosage of oral terbutaline was 10 mg, which is the in-between dose in relation to the accumulated daily dose, 15 mg, as recommended by the manufacture, and the 7.5 mg that is given per administration.

The experimental protocol consisted of two trials that were separated by at least 1 week to ensure complete washout of terbutaline (Borgström et al., 1989). At the first trial, 10 mg terbutaline was administrated orally as two tablets, and at the second trial, 4 mg inhaled dry powder terbutaline was administrated as 8 puffs as a single dose (0.5 mg/puff). Subjects consumed 200 ml water after administration. The inhalation procedure was trained with all participants before inhalation of the drug. Prior to administration of terbutaline, subjects received a standardized meal of 2 cc (85 g) oatmeal, a teaspoon of white sugar and 500 ml partly skimmed milk (total 2.377 kJ) with 300 ml of water. 30 min later the drug was administrated. After administration of study drugs, subjects exercised for 90 min at an intensity corresponding to 65% of their $\dot{\text{V}}{\text{O}}_{2}\text{max}$ (186 ± 11 W) on a bike ergometer. At the end of exercise, subjects received 500 ml of water with protein, as to mimic post-exercise protein substitution. For the next 4½ h, subjects remained inactive.

Six hours after administration of study drugs, subjects were allowed to drink and eat without restrictions.

Blood samples (9 ml) were collected 15, 30, 60, 120, 180, and 240 min after administration of terbutaline. Blood samples were kept at 5°C for 30 min after which they were spun at 3000 rpm for 15 min. Serum was then collected in 2.5 ml cryo tubes and frozen at −80°C until analysis.

Urine samples were collected in 40 ml aliquots 2, 4, 6, 12, and 24 h after administration of terbutaline. The aliquots were immediately frozen and stored at until −80°C analysis. Samples 12 and 24 h after administration were collected by the subjects' and frozen at −20°C until delivery the following day to the department, where they were stored at −80°C.

### Analysis of serum and urine

**Figure 2:**
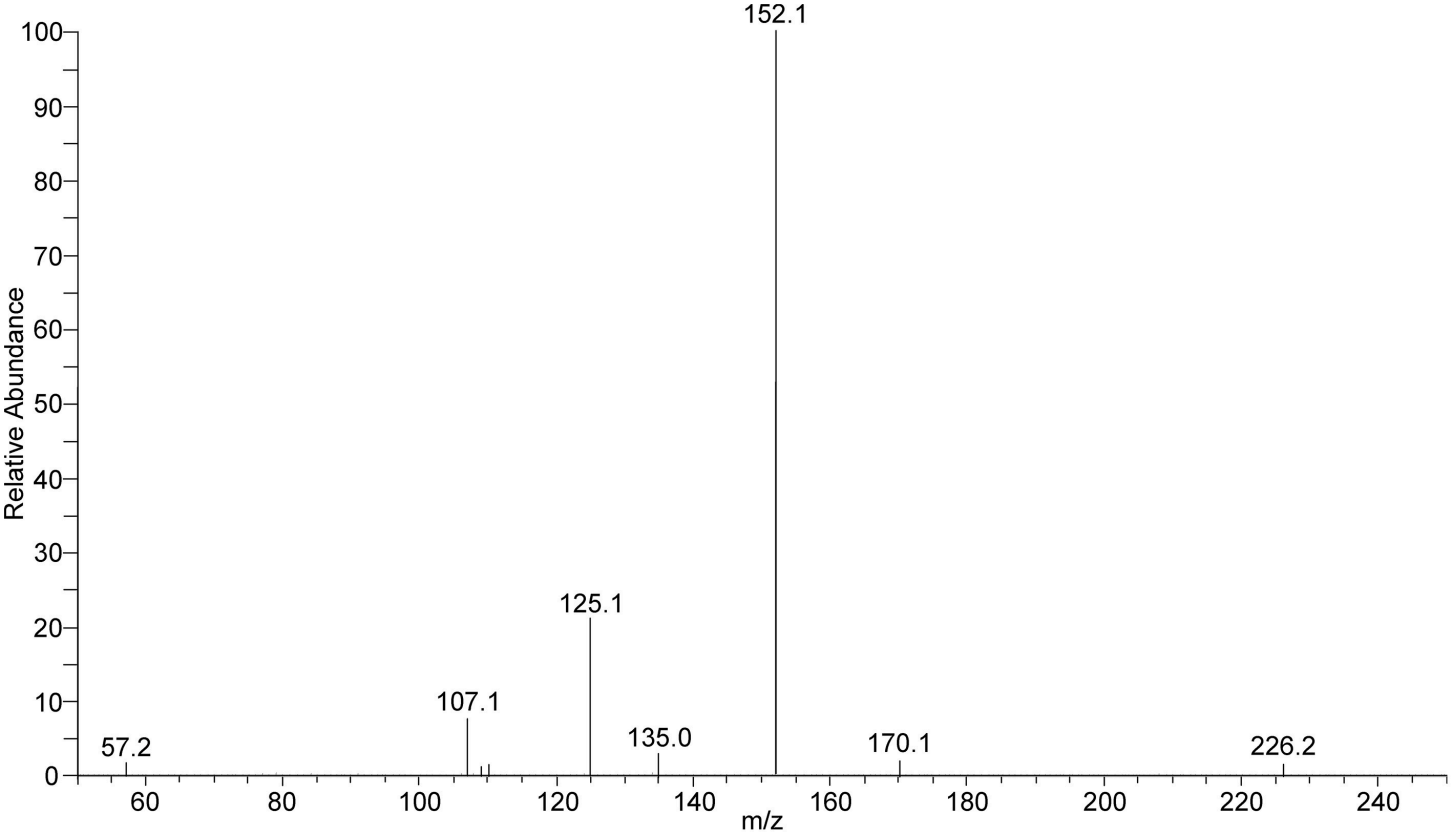
**ESI product ion scan of terbutaline in positive ionization (*m/z* 226 [M+H]^+^, CE 20 V)**.

**Figure 3:**
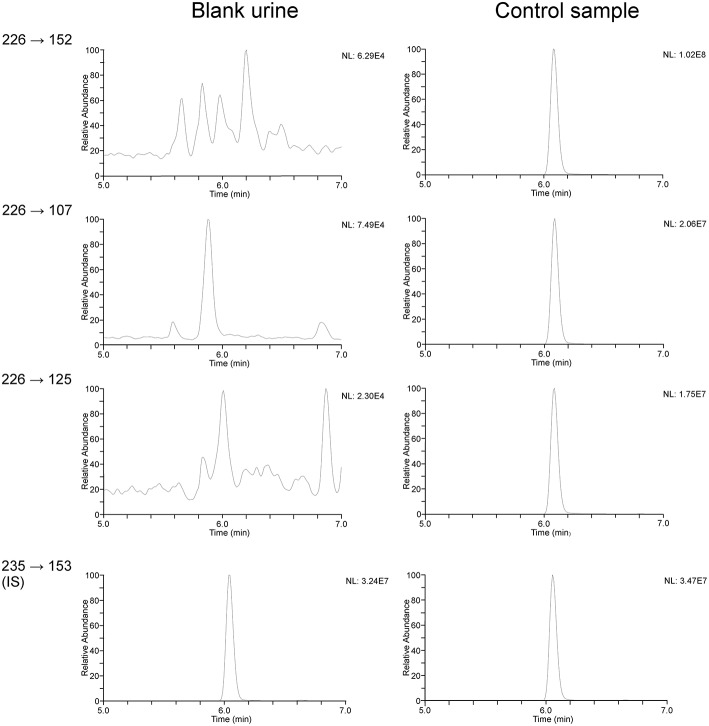
**HPLC-MS/MS chromatograms of terbutaline (*m/z* 226–152, 226–107, and 226–125 [M+H]^+^) and d_9_-terbutaline as internal standard (*m/z* 235–153 [M+H]^+^) in a control sample (500 ng/ml) compared to blank urine**.

Serum and urine samples were analyzed by the WADA accredited Norwegian Doping Control Laboratory in Oslo, Norway. Samples were shipped from Bispebjerg University Hospital, Copenhagen, Denmark on dry ice keeping the temperature at −80°C until arrival at the Norwegian Doping Control Laboratory. Concentrations of terbutaline in serum and urine were quantified by high performance liquid chromatography coupled to tandem mass spectroscopy using a method described in a former study by Elers et al. (2012a) with some minor modifications. In brief, 0.5 ml of urine was diluted with 1.5 ml of deionized water, and 30 μl internal standard solution (d_9_-terbutaline; Sigma, Schnelldorf, Germany, 5 μg/ml in methanol) was added. After addition of 2 M sodium acetate buffer pH 5, 25 μl β-glucuronidase from *E. coli* (Roche Diagnonstics, Mannheim, Germany) was added. Samples were incubated for 1 h at 55°C. Afterwards samples were applied onto Oasis-MCX solid-phase extraction columns (60 mg; Waters, Milford, Massachusetts). The columns were conditioned with 2 ml methanol and afterwards 2 ml deionized water. After loading of the samples, the columns were washed with 2 ml 0.1 M HCl and afterwards with 2 ml methanol. Analytes were eluated with 2 ml NH_3_/methanol (5:95, v/v), and samples were evaporated to dryness. Residues were dissolved in 150 μl water/acetonitrile (95:5, v/v). Sample analysis was performed on a Thermo Surveyor HPLC system coupled to a Thermo TSQ Quantum mass spectrometer (Thermo, San Jose, California). For chromatographic separation a 150 × 1.2 mm, 3 μm Betasil C8 column (Thermo) was used. Analytes were separated using the following gradient of water/acetonitrile (95:5, v/v) with 5 mM ammoniumformiate (A) and water/acetonitril (5:95, v/v) with 5 mM ammoniumformiate (B) at a flow rate of 200 μl/min: 0% B (0 min), 0% B (1 min), 80% B (10 min), 80% B (12 min). The mass spectrometer was operated in positive electrospray ionization mode. ESI product ion scan of terbutaline in positive ionization is illustrated in Figure 2. The following transition reactions were monitored (collision energy): 226.2–152.2 (21 V), 226.2–125.2 (32 V), 226.2–107.2 (41 V) (for terbutaline), and 235.2–153.2 (18 V) (for d_9_-terbutaline; Figure 3). Quantification of terbutaline was performed by using a 5-point calibration curve covering a concentration range from 5 to 1000 ng/ml. Urine samples exceeding 1000 ng/ml were appropriately diluted and reanalyzed. Quantification of serum terbutaline followed the same analytical procedures as described for urine, with some adjustments. The 5-point calibration curve was prepared in Autonorm serum (Sero, Billingstad, Norway), and the calibration range was between 1 and 10 ng/ml. Internal standard addition was adjusted (50 μl d_9_-terbutaline, 0.1 μg/ml in methanol). Residues after solid phase extractions were dissolved in 75 μl water/acetonitrile (95:5, v/v). Serum samples exceeding the range of the calibration curve were appropriately diluted and reanalyzed.

Validation demonstrated suitability of the method in accordance with WADA's technical documents (The World Anti-Doping Agency Laboratory Comittee, 2014).

### Pharmacokinetic calculations

The bioavailability ratio *F*, between oral and inhaled administration of terbutaline was calculated from the total urine excretion of unchanged terbutaline using the following equation (Kampmann et al., 2011):

$$\begin{array}{l} F=\frac{U_{\text{Oral}}^{\infty }}{Dose_{\text{Oral}}}\times \frac{Dos{\text{e}}_{\text{Inhalation}}}{U_{\text{Inhalation}}^{\infty }}\; \end{array}$$

Where *F* is the bioavailability ratio between the two routes of administration, *U*^∞^ is the urine excretion from zero to last sample (24 h after administration) and Dose is the administered dose for the different routes.

The impact of adjusting urine samples for USG on concentrations was determined using the following equation in accordance with WADA technical documents (The World Anti-Doping Agency Laboratory Comittee, 2014):

$$\begin{array}{l} {\text{C}}_{\text{adjusted}}=\text{C}\times \left(0.02∕\left({\text{USG}}_{\text{sample}}-1\right)\right) \end{array}$$

Where the C_adjusted_ is the USG adjusted urine concentration, the USG_sample_ is the USG of the given sample, and C is the raw unadjusted urine concentration.

### Statistics

Statistical analyses were performed in SPSS version 22 (IBM, USA). Sample size was determined for the primary response variable (urine concentration of terbutaline) for a linear mixed model repeated measures design. Effect size and standard deviation were estimated from previous pharmacokinetic studies of β_2_-agonists (Elers et al., 2012a; Haase et al., 2015).

The data were tested for normality using the Shapiro–Wilks test and Q-Q plots and are presented as means ± standard error of the mean (SEM). To estimate changes in urine concentrations with the intervention, a three factorial linear mixed model was used with USG, trial, and sampling time as fixed effects and subjects as a random factor. To estimate changes in urine excretion and serum concentrations with the intervention, a two factorial linear mixed model was used with trial and sampling time as fixed effects and subjects as a random factor. In case of multiple comparisons, a Bonferroni correction was used as a post hoc test. Differences in C_max_, T_max_, and AUC was determined with a *t*-test. Pearson's bivariate test was used to test correlation between USG and unadjusted urine concentrations. Level of significance was set to *P* ≤ 0.05.

## Results

### Subjects and side effects

Twelve out of 14 subjects completed the study. Two subjects were excluded due to social reasons leading to non-compliance with the protocol. Terbutaline was well-tolerated regardless the route of administration and only minor side effects were reported by the subjects, including tachycardia (*n* = 4) after inhalation of 4 mg of terbutaline.

### Serum concentrations of terbutaline

**Figure 4:**
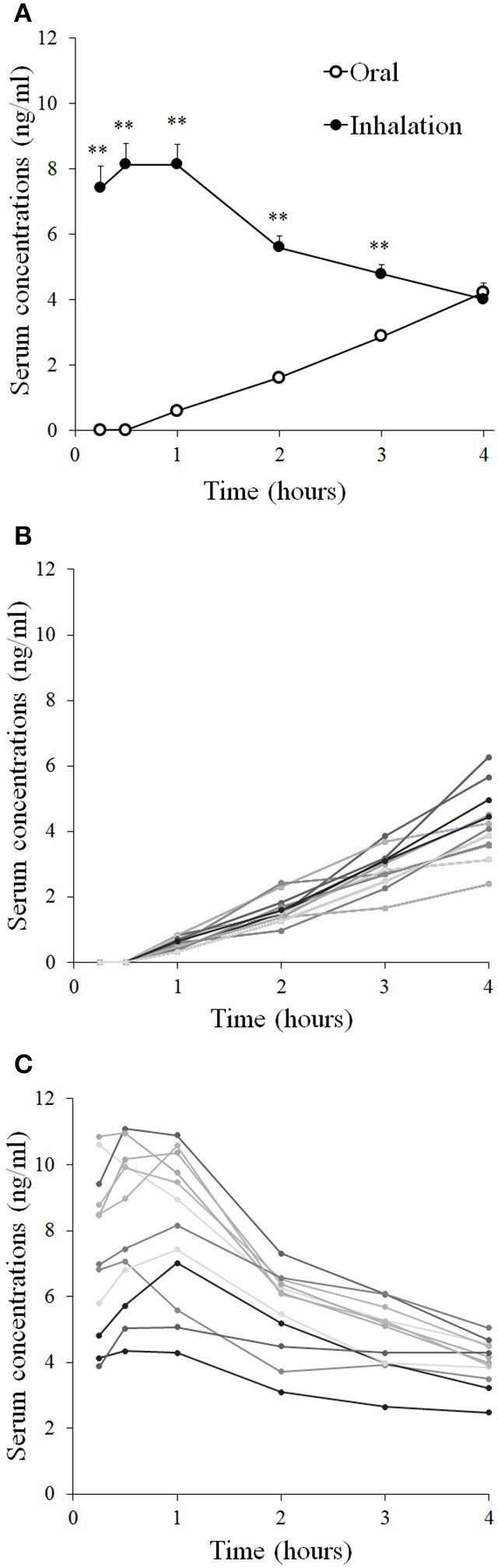
**Serum concentrations of terbutaline after oral administration of 10 mg and inhaled administration of 4 mg in healthy trained men (*n* = 12)**. Mean serum concentrations ± SEM **(A)**, individual concentrations after oral administration **(B)**, and inhalation **(C)**. ^**^Different (*P* ≤ 0.01) from oral.

Serum concentrations of terbutaline after inhaled and oral administration are presented in Figures 4A–C. Serum concentrations of terbutaline rose faster after inhaled administration compared to oral. Observed serum C_max_ and AUC within the first 4 h after administration were higher (both *P* ≤ 0.001) after inhaled administration compared to oral (Table 2). However, as illustrated in Figure 4A, serum concentrations had not reached their peak 4 h after oral administration.

**Table 2:** **Pharmacokinetic parameters**.

	**Oral**	**Inhaled**
Observed AUC_(0−4h)_(ng/ml × min)	422 ± 22	1308 ± 119^**^
Observed C_max(0−4h)_(ng/ml)	4.2 ± 0.3	8.5 ± 0.7^**^
Observed T_max(0−4h)_ (min)	240 ± 0	44 ± 5^**^
Urine excretion (μg)	303 ± 43	378 ± 30

AUC, Area Under the Curve; C_max_, Maximal Serum Concentration; T_max_, Time to maximal Concentration.

** Different (P ≤ 0.01) from oral.

### Urine concentrations of terbutaline

**Figure 5:**
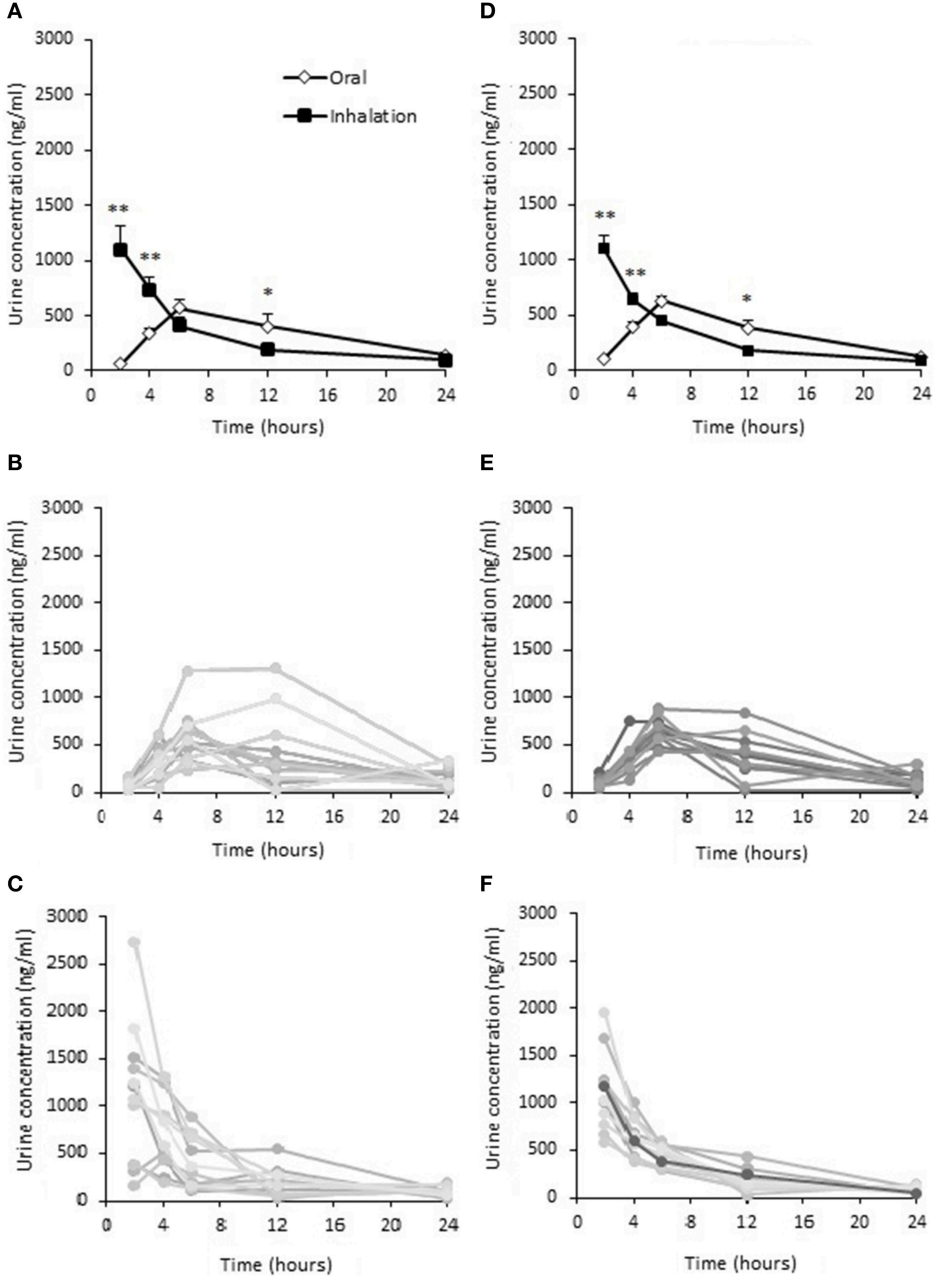
**Urine concentrations of terbutaline after oral administration of 10 mg and inhaled administration of 4 mg in healthy trained men (*n* = 12)**. Mean ± SEM urine unadjusted **(A)** and adjusted **(D)** concentrations, individual concentrations after oral administration **(B)** and inhalation **(C)**. Adjusted mean urine concentrations **(D)**, individual concentrations after oral administration **(E)** and inhalation **(F)**. ^*^Different (*P* ≤ 0.05) from oral. ^**^Different (*P* ≤ 0.01) from oral. Values are means ± SEM **(A,D)** and measured values **(B–E)**.

Urine concentrations of terbutaline after inhaled and oral administration are presented in Figures 5A–F. Urine concentrations of terbutaline were higher (*P* ≤ 0.001) after inhalation compared to oral administration 2 and 4 h after administration (Figure 5D), whereas concentrations were lower (*P* ≤ 0.05) for inhalation than oral administration 12 h after administration (Figure 5A). After oral administration, the highest individual urine concentration was observed 12 h after administration reaching 1308 ng/ml when unadjusted for USG, whereas it was 2736 ng/ml 2 h following inhalation. If adjusted for USG, the highest individual urine concentration was observed 6 h after oral administration, reaching 881 ng/ml, whereas it was observed 2 h after inhalation, reaching 1954 ng/ml.

### Urine excretion of terbutaline

**Figure 6:**
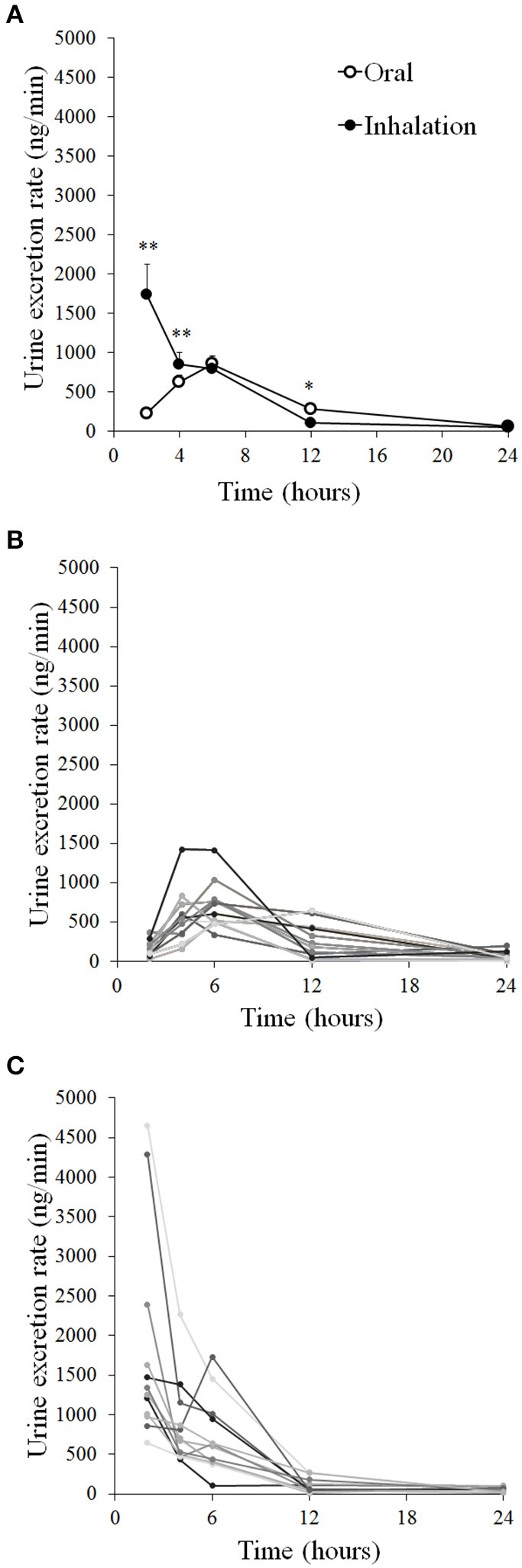
**Mean urine excretion rates of terbutaline after oral administration of 10 mg and inhaled administration of 4 mg in healthy trained men (*n* = 12)**. **(A)** Individual excretions after oral administration **(B)** and inhalation **(C)**. ^*^Different (*P* ≤ 0.05) from oral. ^**^Different (*P* ≤ 0.01) from oral. Values are means ± SEM **(A)** and measured values **(B,C)**.

Urine excretion rate of terbutaline after oral and inhaled administration is presented in Figures 6A–C. Urine excretion rate of terbutaline was higher after inhalation compared to oral 2 and 4 h after administration (*P* ≤ 0.001), whereas excretion rate was lower for inhalation than oral administration 12 h after administration (*P* ≤ 0.05). Total amount of terbutaline excreted during the 24 h after administration was not different between oral administration of 10 mg and inhaled administration of 4 mg (*P* = 0.20; Table 2). From the total urine excretion, the bioavailability ratio was 3.8: 1 (inhaled: oral; *P* ≤ 0.001).

## Discussion

The main finding of this study was that the peak urine concentrations of terbutaline were higher after inhaled administration of 4 mg than oral administration of 10 mg terbutaline. For doping control purposes, the present findings thus indicate that it is not possible to discriminate prohibited oral ingestion of terbutaline from inhaled administration on the basis of the concentration of terbutaline (sum of the free drug and the glucuronide) in urine samples in relation to WADA's prohibited list (The World Anti-Doping Agency, 2016).

In comparison with other pharmacokinetic studies of terbutaline (Nyberg and Kennedy, 1984; Borgström et al., 1989; Schmekel et al., 1992; Elers et al., 2012a), our study is the first to investigate the pharmacokinetics of terbutaline during a setup applicable for training and exercise with respect to doping control analysis. Despite the 2.5-fold lower nominal dose of inhaled terbutaline compared to oral in the present study, we observed that the urine concentrations of terbutaline were higher during the first 8 h for inhaled administration than oral. This apparent difference between inhaled terbutaline and oral ingestion is possibly related to differences in bioavailability between the two routes of administration. Indeed, we observed that serum AUC of terbutaline was higher for 4 mg inhaled terbutaline than 10 mg of oral terbutaline. This observation is in accordance with Elers et al. (2012a), in which inhalation of 2 mg terbutaline resulted in similar peak serum concentrations as 10 mg oral ingestion 0–6 h following administration. In the present study, it could be speculated that calculation of serum AUC between the routes of administration was limited by a too short period of blood sampling (0–4 h following administration). Indeed, we failed to observe a peak for serum concentrations of terbutaline after oral administration in some of the subjects. Nyberg et al. (Nyberg and Kennedy, 1984) observed a Tmax of 4 h after administration of slow-release 7.5 mg tablets of terbutaline. However, we observed that the bioavailability ratio, based on urine excretion 0–24 h after administration, was 3.8: 1 for inhaled versus oral administration of terbutaline, clearly indicating a pronouncedly higher systemic bioavailability for the inhaled route.

The markedly lower systemic bioavailability of orally administered terbutaline compared to inhaled is likely attributed to the first pass metabolism, that oral substances undergo. Another factor that could explain the differences between the two routes is exercise. It is well-known, that distribution of cardiac output changes during exercise, in which contracting skeletal muscle is prioritized over the splanchnic region leading to a reduction in blood flow through the gastrointestinal system (Wade et al., 1956; Williams and Leggett, 1989; Perko et al., 1998). Exercise could as such reduce gastrointestinal absorption of oral terbutaline. Indeed, Elers et al. (2012a) observed a higher C_max_ after oral administration of 10 mg than that observed in the present, thus indicating that the amount absorbed is reduced by exercise (Elers et al., 2012a). On the other hand, Schmekel et al. (1992) found C_max_ after inhalation to be higher after exercise compared to resting, which is consistent with our results compared to Elers et al. (2012a). Furthermore, exercise increases pulmonary blood flow, which may increase absorption of terbutaline when administered by inhalation (Musch et al., 1987; Schmekel et al., 1992). Accordingly, we observed a faster T_max_ following inhalation of terbutaline than that observed by Elers et al. (2012a) in resting subjects. This is also consistent with Schmekel et al. in which T_max_ was 1 h following inhalation at rest, whereas T_max_ was 30 min during exercise (Schmekel et al., 1992). Thus, T_max_ and C_max_ seem to be prolonged and lowered, respectively, by exercise after oral administration, whereas it is faster and higher after inhalation. Despite the different collection time points, we found almost the same urine concentrations after oral administration as those observed by Elers et al. and Nyberg et al. (Nyberg and Kennedy, 1984; Elers et al., 2012a) with higher urine concentrations after inhalation, supporting a higher uptake of inhaled terbutaline (Elers et al., 2012a). In relation to urine excretion only Nyberg et al. (Nyberg and Kennedy, 1984) reported excretion rate which compared to ours was lower when adjusted for dose.

In the present study, we observed no differences between USG adjusted and unadjusted urine concentrations of terbutaline, which was opposite to the results obtained by Elers et al. (2012a) when they examined terbutaline at rest. Hostrup et al. (2012) has shown that urine samples unadjusted for USG can result in false positive or false negative outcomes especially in highly concentrated urine samples of dehydrated subject. Nonetheless, our results showed no difference, which indicate that the subjects were not sufficiently dehydrated to affect the USG significantly. Therefore, rehydration during exercise plays a crucial role for USG and false positive outcomes in doping analysis.

Terbutaline is a commonly prescribed drug in asthma treatment and athletes are allowed to use it in and out of competition with a TUE. Given the current WADA regulations for β2-agonists, it is important to be able to distinguish between routes of administration of terbutaline, because oral use is prohibited (The World Anti-Doping Agency, 2016). This study was, to our knowledge, the first to investigate the oral and inhaled route in relation to exercise. As terbutaline often is used in conjunction with exercise and competitions, the present setup is more applicable than previous studies conducted at rest. Our main findings show, that the routes of administered terbutaline cannot be distinguished. However, it could be speculated that the route is not important in relation to doping. It could as such be debated whether or not the discrimination of prohibited or allowed use of terbutaline should be set on the basis of administered dosage and not on the basis of administered route since the athlete achieve higher systemic concentrations after inhalation compared to oral. Thus, it is more important to look at the administered dose in relation to doping because both oral administration and high dose inhalation of terbutaline may increase maximal sprint ability and muscle force (Hostrup et al., 2014a,b; Kalsen et al., 2016). In order to discriminate therapeutic inhaled use of terbutaline from prohibited supratherapeutic use as done for salbutamol, formoterol, and salmeterol (The World Anti-Doping Agency, 2016) a general urinary threshold for terbtualine is warranted. In any case, this should be investigated further in future studies. While the dosing regimen of 8 × 0.5 mg terbutaline by inhalation as a single dose administered in the present study is higher than normal therapeutic recommendation it does not exceed the maximal daily dose as stated in the manufacturers summary of product characteristics. Furthermore, the dose is equipotent to the maximal allowed dose (8 × 0.2 mg) for inhaled salbutamol on the WADA-prohibited list (The World Anti-Doping Agency, 2016). Given the potential of terbutaline as a performance enhancing drug, a urinary threshold should be defined for terbutaline based on urine concentrations observed after inhalation. We suggest, that the dose of terbutaline to be investigated should be 8 × 0.5 mg, as this both includes the highest allowed dose, as well as the majority of inter-individual variations there might be, minimizing the risk of false positive AAFs, as has been done for the other β2-agonists on WADA's prohibited list (The World Anti-Doping Agency, 2016).

In summary, the present study shows that it is not possible to discriminate 4 mg inhaled terbutaline from 10 mg oral administration of terbutaline based on urine concentrations of terbutaline (sum of the free drug and the glucuronide) for doping control purposes. This is likely related to the markedly higher systemic bioavailability for inhaled compared to oral administration of terbutaline. Given these results, any potential urine threshold for terbutaline should be based on the systemic response rather than the route of administration. With the current regulations, athletes that have acquired a TUE for terbutaline, may potentially inhale very high doses and enhance performance. This advantage could be eliminated if a general urinary threshold limit based on both pharmacokinetics and–dynamics is introduced.

## Author contributions

The experiment was performed at the Respiratory Research Unit, Bispebjerg University Hospital and samples were analyzed at the Norwegian Doping Control Laboratory, Oslo University Hospital. All authors contributed to the conception and design of the experiment, collection, analysis and interpretation of data, and drafting the manuscript or revising it critically for important intellectual content. All authors approved the final version of the manuscript.

## Funding

The study was performed with financial support from the World Anti-Doping Agency.

### Conflict of interest statement

The authors declare that the research was conducted in the absence of any commercial or financial relationships that could be construed as a potential conflict of interest.
